# Proteomic signatures of acute oxidative stress response to paraquat in the mouse heart

**DOI:** 10.1038/s41598-020-75505-8

**Published:** 2020-10-28

**Authors:** Vishantie Dostal, Silas D. Wood, Cody T. Thomas, Yu Han, Edward Lau, Maggie P. Y. Lam

**Affiliations:** 1 Department of Medicine, Division of Cardiology, University of Colorado Anschutz Medical Campus, Aurora, CO 80045 USA; 2 Department of Cardiology, University of Colorado Anschutz Medical Campus, Aurora, CO 80045 USA; 3 Department of Biochemistry and Molecular Genetics, University of Colorado Anschutz Medical Campus, Aurora, CO 80045 USA; 4 Consortium for Fibrosis Research & Translation, University of Colorado Anschutz Medical Campus, Aurora, CO 80045 USA

**Keywords:** Proteomics, Cardiovascular biology

## Abstract

The heart is sensitive to oxidative damage but a global view on how the cardiac proteome responds to oxidative stressors has yet to fully emerge. Using quantitative tandem mass spectrometry, we assessed the effects of acute exposure of the oxidative stress inducer paraquat on protein expression in mouse hearts. We observed widespread protein expression changes in the paraquat-exposed heart especially in organelle-containing subcellular fractions. During cardiac response to acute oxidative stress, proteome changes are consistent with a rapid reduction of mitochondrial metabolism, coupled with activation of multiple antioxidant proteins, reduction of protein synthesis and remediation of proteostasis. In addition to differential expression, we saw evidence of spatial reorganizations of the cardiac proteome including the translocation of hexokinase 2 to more soluble fractions. Treatment with the antioxidants Tempol and MitoTEMPO reversed many proteomic signatures of paraquat but this reversal was incomplete. We also identified a number of proteins with unknown function in the heart to be triggered by paraquat, suggesting they may have functions in oxidative stress response. Surprisingly, protein expression changes in the heart correlate poorly with those in the lung, consistent with differential sensitivity or stress response in these two organs. The results and data set here could provide insights into oxidative stress responses in the heart and avail the search for new therapeutic targets.

## Introduction

Oxidative stress is widely implicated in cardiovascular disorders and other age-related diseases^1,2^. Evidence of oxidative stress is commonly found in many animal models of heart diseases, but antioxidant therapeutics have only seen limited success and were in some cases harmful, suggesting further work is needed to understand stress response pathways and identify new therapeutic targets^3,4^. It is now known that acute oxidative stress triggers complex and multifactorial changes to the cardiac proteome including widespread chemical and enzymatic oxidative modifications^5,6^ as well as the activation of integrated stress response pathways^7^. Prior proteomics data on the global responses of cultured mammalian cells to hydrogen peroxide in vitro pointed to close connections between oxidative stress and the unfolded protein response, as well as a diverse number of regulatory pathways involved in metabolism and protein translation^8^. Whether similar proteome-wide responses are recapitulated in the heart of in vivo animal models remains unclear.

Despite the biological significance of oxidative stress to heart diseases, there have been few reports on the effect of direct acute oxidant exposure on protein abundance in the heart. To gain insights into how acute oxidative stress remodels the heart proteome, we examined the effect of acute exposure to paraquat as a model of oxidative stress in the mouse. Paraquat (*N*,*N*$^\prime$-dimethyl-4,4$^\prime$-bipyridinium dichloride) is a powerful pro-oxidant that generates mitochondrial reactive oxygen species in situ at the respiratory chain^9–11^. In humans, chronic environmental exposure to residues of paraquat used as pesticide has been linked to the development of Parkinson′s disease^12^, whereas in rare cases acute paraquat poisoning at higher dose has also been reported that leads to significant multi-organ toxicity and mortality^13,14^. In laboratory experiments, high doses of paraquat ($\sim$ 20–80 mg/kg) have been broadly used as in rodents to reliably induce acute oxidative stress in the heart^15–18^ and in the lung^19,20^. In this study, we compared normal and acute paraquat-stressed mouse hearts using quantitative mass spectrometry, with the goal of assessing the effect of acute oxidative damage on the cardiac proteome.

## Results

### Moderate to high doses of paraquat induces acute oxidative stress responses in the heart

**Figure 1:**
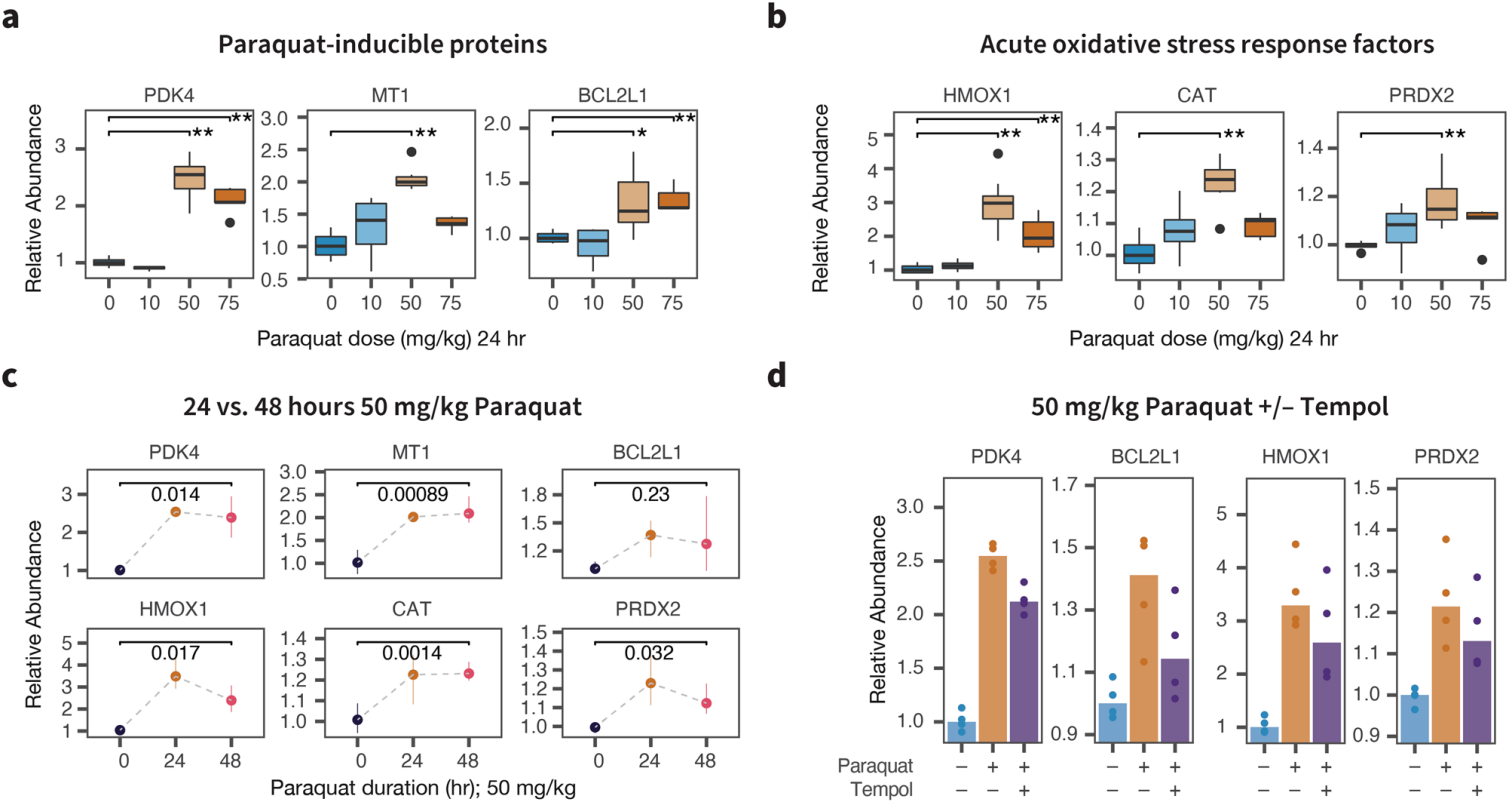
Moderate and high doses of paraquat induce acute oxidative stress response proteins in the heart. (**a**) Moderate and high doses of paraquat for 24 h led to an increase in protein abundance of known paraquat-induced genes in the hearts including PDK4, MT1, and BCL2L1; $$^*$$t-test $$\hbox {P} < 0.05$$; $$^{**}$$0.005 vs. vehicle. (**b**) Moderate and high doses of paraquat for 24 h led to a robust increase in heme oxygenase 1 (HMOX1) as well as a moderate increase in the protein level of other NRF2-induced acute oxidative stress response elements in the heart; $$^*$$t-test $$\hbox {P} < 0.05$$; $$^{**}$$0.005 vs. vehicle. (**c**) A number of induced proteins remained elevated at 48 h after the paraquat dose including PDK4, MT1, and CAT, whereas other acute response elements including HMOX1 began to subside; numbers: t-test P values between 48 h vs. vehicle. (**d**) Pre-treatment of the anti-oxidant compound Tempol partially repressed the up-regulation of paraquat-induced and acute oxidative stress response proteins.

Paraquat is a mitochondria-targeted redox-cycler that acts as a superoxide generator to produce ROS through interactions with Complex I within the inner mitochondrial matrix^11^. To evaluate the proteomic responses to elevated oxidative stress, we first administered various doses of paraquat to C57BL/6J mice for 24 h. The animals (n = 4 per group) were exposed to three doses of paraquat (low—10 mg/kg; moderate—50 mg/kg; and high—75 mg/kg). The doses were chosen from a range previously reported for cardiac research models in the literature, with reports showing that exposure at 40–80 mg/kg for up to 48 hours induced acute oxidative stress responses as well as reduced contractile function and calcium handling in the mouse heart^15,18,21–23^. We first compared whether the applied paraquat doses elevated protein abundance of previously documented paraquat induced genes in the heart using mass spectrometry (Fig. 1a). Pyruvate dehydrogenase kinase 4 (PDK4) is a potent inhibitor of pyruvate conversion into acetyl-CoA that reduces oxygen consumption in the respiratory chain and has previously been found to be strongly induced by 50 mg/kg paraquat^24^ as well as the related dipyridyl diquat^25^. Under moderate to high doses but not the low dose of paraquat, we observed a strong induction of PDK4, in addition to the products of two other known paraquat-induced genes in the heart, metallothionein-1 (MT1) and BCL2-like 1 (BCL2L1).

We next compared whether the paraquat doses employed in the experiment activated oxidative stress responses in the heart as expected. In the heart and in other organs, acute oxidative stress response is under the master regulatory control of the nuclear respiratory factor 1/2 (NRF1/2) pathway^26,27^. Under physiological conditions, NRF2 is continuously degraded and has a short half-life, but under stress conditions NRF2 accumulates and translocates into the nucleus to activate genes with antioxidant response element (ARE) sequences and thereby orchestrates the acute phase of cellular oxidative stress responses. At 24 h, we observed a robust induction of heme oxygenase 1 (HMOX1), a primary cardioprotective oxidative stress response protein in the heart (Fig. 1b). At the same time, other NRF2-induced factors were modestly elevated including catalase (CAT), glutathione dismutase 1 (GPX1), and glutamate-cysteine ligase (GCLC/GCLM) (Supplementary Fig. S1a). In our experiment, the induction of stress response genes was the highest at 50 mg/kg, hence we selected this dose to further assess the effect of exposure duration and co-administered antioxidant. Contrasting the effects at 24 vs. 48 h of paraquat exposure, we further found that the assessed paraquat signatures PDK4, MT1, and BCL2L1 remained elevated at 48 h after paraquat exposure, but several NRF2-induced oxidative response proteins regressed partially toward the baseline, with the exception of catalase, suggesting the acute phase response may begin to subside 48 h after paraquat administration (Fig. 1c; Supplementary Fig. S1b). Pre-treatment with Tempol (2 mM; 24 h) prior to 50 mg/kg paraquat exposure for 24 h partially reverted the oxidative stress response and paraquat-induced proteins toward the baseline, consistent with the observed protein changes arising from bona fide oxidative stress response induced in the animal models (Fig. 1d; Supplementary Fig. S1c; also see section on Responses to Antioxidant Treatments).

### Compartment-specific proteome response to acute oxidative stress

**Figure 2:**
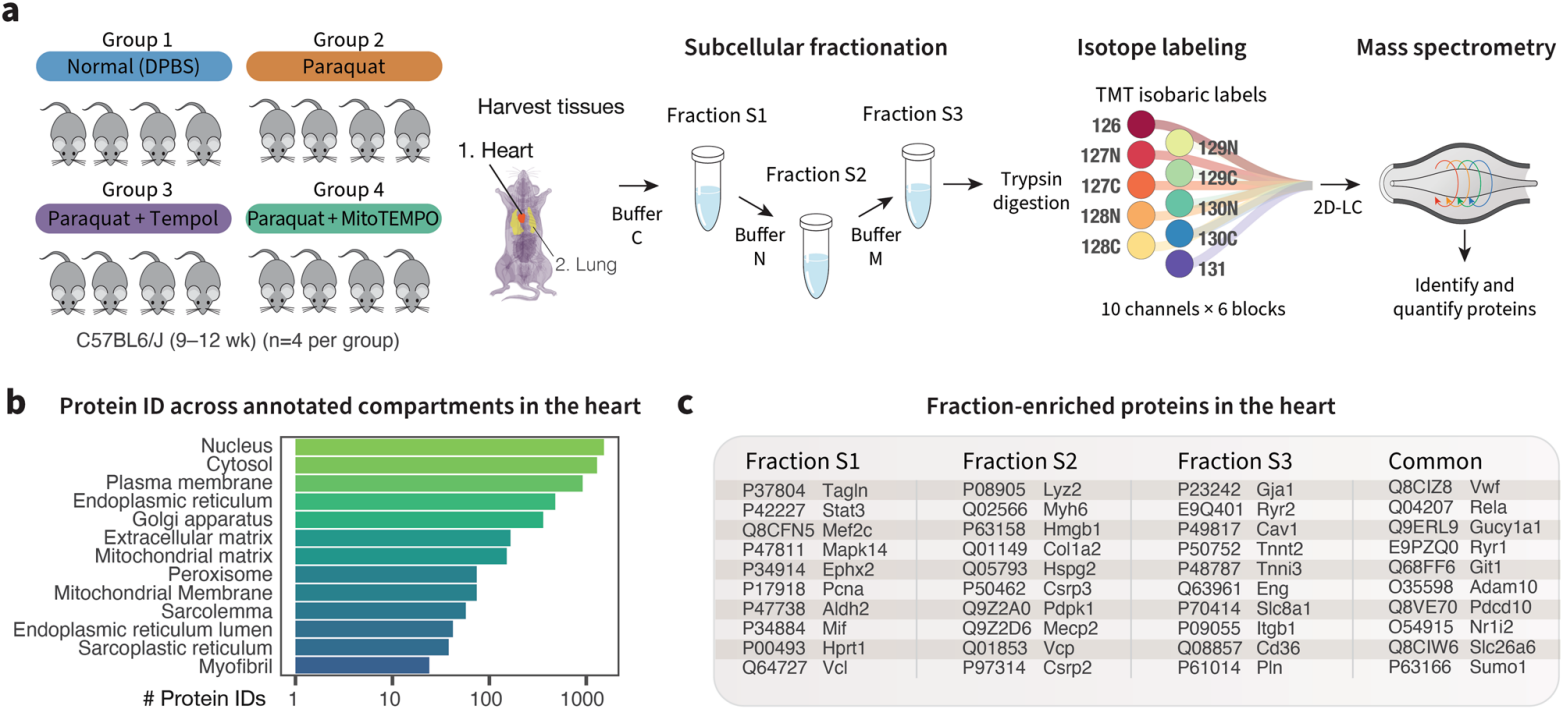
Mass spectrometry analysis of protein expression in normal mouse hearts and following acute paraquat exposure. (**a**) Experimental design. C57BL/6J mice (n = 4 per group) were treated with a vehicle (DPBS) or a moderate dose (50 mg/kg) of paraquat for 24 h. Additional groups were given the anti-oxidants Tempol and MitoTEMPO. Cardiac tissues were harvested, homogenized and further separated into three subcellular fractions (S1, S2, S3) using commercially available differential lysis buffers. The resulting proteins were digested and labeled with isotope tags, then combined for protein identification and quantification. (**b**) Number of proteins identified in the proteomics experiment across selected cellular compartments. (**c**) The subcellular fractions are enriched in different cellular compartments and proteins. Selected proteins relevant to cardiovascular research that are highly enriched in each fraction ($\ge$ twofold, limma adjusted $\hbox {P} \le 0.01$) or are shared across all fractions are shown.

The initial results above confirmed that exposure to moderate to high doses (50–75 mg/kg) of paraquat for 24–48 h robustly activated classic acute oxidative stress responses in the mouse heart, and at the same time induced other known paraquat-induced signatures. To further investigate how acute stress responses remodel the global cardiac proteome, we selected the medium dose paraquat exposure (50 mg/kg, 24 h) and acquired deep quantitative proteome profiles using subcellular fractionation and two-dimensional peptide separation (Fig. 2a). To improve coverage, we used a commercial differential lysis workflow to perform subcellular fractionation on the harvested tissue which created three distinct fractions (S1, S2, and S3) containing sets of proteins distinguished by localization and solubility. The samples from each treatment group, tissue, and fraction were randomized and labeled with stable isotopes for quantitative mass spectrometry (Supplementary Fig. S2a–d). From the isobaric-labeled quantitative proteomics data, we quantified 5323 non-redundant protein groups to compare the expression of protein groups in the hearts of vehicle vs. paraquat treated mice (Supplementary Table S1). The proteomics experiment covered multiple cellular compartments (Fig. 2b). We compared the fraction compositions and found distinct sets of proteins enriched in each fraction (Fig. 2c). Gene annotations of enriched proteins showed that the S1 fraction is enriched in cytosolic components, whereas both the S2 and S3 fractions are enriched in mitochondria and membrane proteins over the S1 fraction (Supplementary Fig. S3a). When comparing the S2 and S3 fractions directly, we further found that the S2 fraction is relatively enriched in myofibrils and nuclear proteins whereas the S3 fraction is enriched in endoplasmic reticulum and sarcolemmal proteins (Supplementary Fig. S3b) and overall has more hydrophobic proteins (Supplementary Fig. S3c), suggesting the subcellular fractionation was effective in separating the cardiac proteomes into distinct fractions.

**Figure 3:**
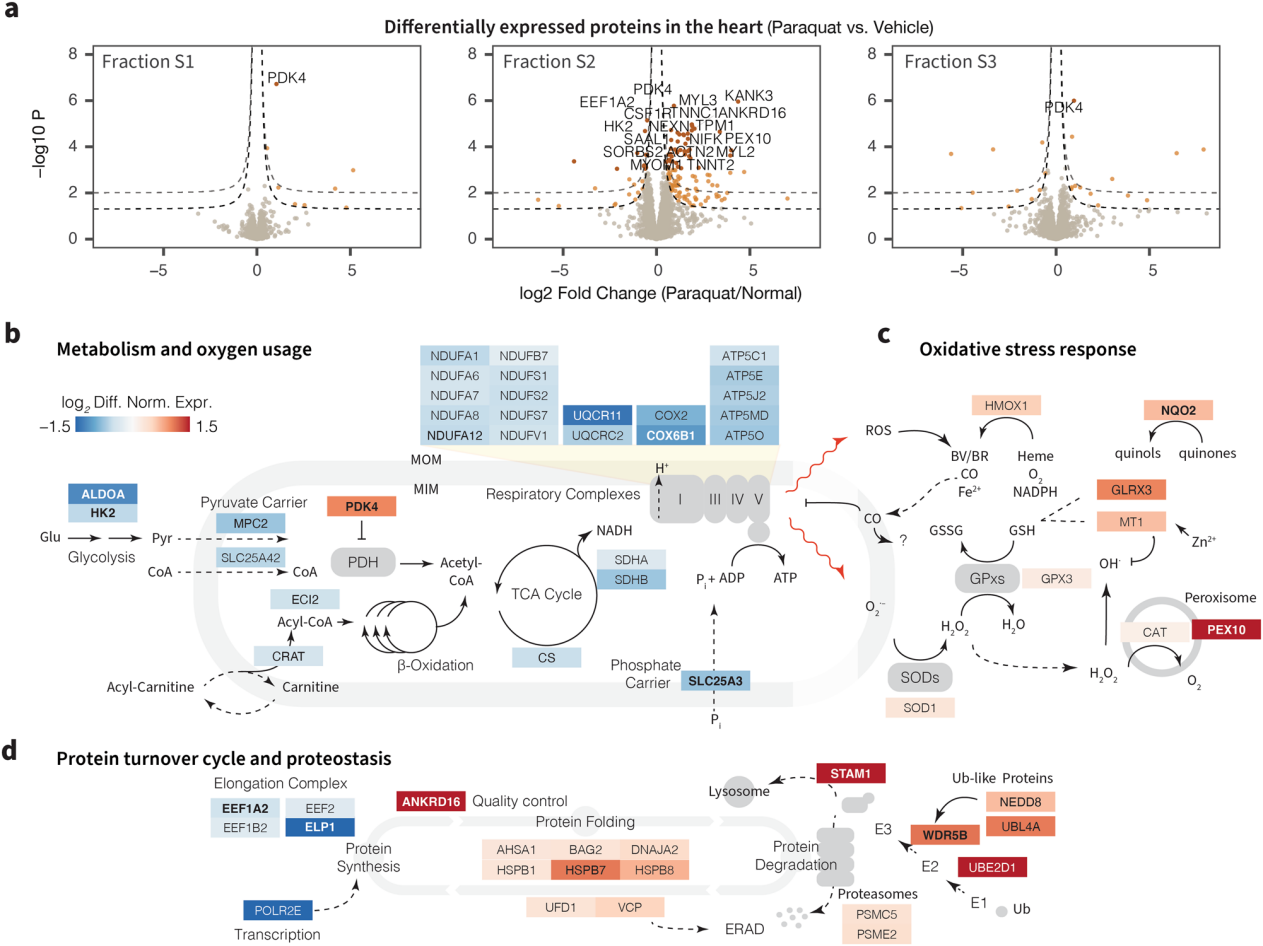
Proteomic signatures of acute oxidative stress in the heart. (**a**) Paraquat preferentially causes the differential expression of proteins in the S2 fraction, which is enriched in proteins in the mitochondria, endoplasmic reticulum, and nucleus. Dashed lines: nominal significance at absolute logFC of 0.5 and $\hbox {P} \le 0.05$ and $\le 0.01$, respectively. Brown data points are significantly differentially expressed at 10% false discovery rate; up to 30 differentially expressed proteins are labeled. The pathway diagrams illustrate the differential expression of proteins involved in (**b**) central metabolism and respiration, (**c**) oxidative damage response, and (**d**) protein turnover and proteostasis. Colors represent fold change in paraquat vs. normal hearts, proteins labeled in bold correspond to proteins within the 10% false discovery rate filter in the panels above. The proteomic profiles in stressed hearts are indicative of decreased respiration and protein synthesis coupled with increases in antioxidant proteins and protein degradation amid acute paraquat challenge.

Surprisingly, we found that 50 mg/kg paraquat primarily induced differential expression of proteins localized in Fraction S2, with far fewer differentially regulated proteins in fractions S1 and S3 (Fig. 3a). The quantified proteins span diverse cellular pathways, with a total of 1406 Gene Ontology Biological Process (BP) terms, 489 Molecular Function (MF) terms, and 406 Cellular Component (CC) terms represented by $\ge 5$ proteins. Out of these annotations, 23 BP terms had over 100 proteins quantified (Supplementary Fig. S4a) including transport (512), oxidation–reduction processes (325), translation (233), and proteolysis (151). Among differentially expressed proteins, 35 processes were represented by 5 or more proteins (Supplementary Fig. S4b) with notable coverage in oxidative-reduction process (18), muscle contraction (9) and lipid metabolic process (9) terms. Upon closer inspection of the data, we found that acute paraquat exposure broadly induced the differential regulation of a number of mitochondrial and endoplasmic reticulum proteins in the S2 fraction. In particular, we found that the primary feature of the paraquat remodeled proteome appears to be a broad down-regulation of mitochondrial central metabolism proteins, including components of the respiratory chain and the tricarboxylic acid cycle (Fig. 3b). Concomitant with the downregulation of metabolic proteins is a conspicuous up-regulation of PDK4, which serves to inhibit pyruvate influx into the mitochondrial respiratory chain, suggesting that a principal response of the heart to acute oxidative stress is in the suppression of central metabolism and reduction of respiratory oxygen usage.

At the same time, we observed prominent changes in oxidative stress response proteins (Fig. 3c). As above, HMOX1 is induced following paraquat exposure. HMOX1/HO-1 is an inducible isoform of heme oxygenase and a prominent NRF2-dependent factor in oxidative stress response both in the heart and in other tissues under general oxidative stress conditions^28^. HMOX1 degrades heme into iron, CO, and biliverdin, the latter being an antioxidant that scavenges peroxyl radicals^29^. MT1 is also upregulated across all examined fractions. Metallothioneins are efficient chelators of zinc and other heavy metal ions and serve as efficient scavengers of hydroxyl and superoxide radicals^30^. Thirdly, we found evidence of a coordinated proteostasis response through a reduction in global protein synthesis and promotion of protein folding and protein degradation (Fig. 3d). A number of elongation complex proteins including EEF1A2 and EEF2 are down-regulated, whereas protein quality control factors are up-regulated. Among the induced chaperones is HSPB7, a cardiac heat shock protein that is linked to cardiomyopathy in genome-wide association studies and significantly co-occurs with heart failure publications in the literature. However, not every integrated stress response component showed evidence of regulation from the differential expression data. For instance, we did not observe clear trends for differential regulation in mitochondrial import which forms an integral part of the mitochondrial unfolded protein response. Gene annotation term enrichment analysis corroborates that differentially expressed proteins preferentially associate with myofibril components (likely due to S2/S3 transitions, see below), as well as biological processes including fatty acid beta-oxidation (P: 5.7e−3, adjusted P: 0.039) and response to hydroperoxide (P: 6.0e−3, adjusted P: 0.039) (Supplementary Fig. S4c).

Lastly, the proteomics data also uncovered the differential expression of a number of proteins with still unclear functions in the heart, implicating a potential function of these proteins in acute oxidative stress responses. For example: (i) Proline rich protein 16 (PRR16) is significantly activated after paraquat (logFC: 1.77, P: 1.3e−4; adjusted P: 0.019). Promoter binding analysis provides supporting evidence that the *PRR16* gene may be a target of CREB which is activated by ER stress to trigger unfolded protein response. Although our experiment did not identify CREB1, CREB coactivator CRTC2 is also robustly increased (logFC: 1.06, P: 3.4e−3, adjusted P: 9.9e−2), suggesting PRR16 may be a downstream ER stress response factor. The function of PRR16 in the heart is unclear. (ii) Aldehyde dehydrogenase 16 family member A1 (ALDH16A1) is nominally up-regulated under paraquat exposure (logFC: 1.47, P: 2.0e−2; adjusted P 2.5e−1). Although a member of the aldehyde dehydrogenase family, ALDH16A1 contains aldehyde dehydrogenase domains that are thought to be inactive^31^, and its function in the heart remains unknown. It is ubiquitously expressed and has no clear co-expressed genes across human tissues, but previous ChIP-seq data has shown that its promoter region may bind to STAT3^32^, which is also upregulated in the data (logFC: 0.65, P: 3.8e−4; adjusted P: 2.9e−2). (iii) Signal transducing adaptor molecule (SH3 domain and ITAM motif) 1 (STAM) is conspicuously up-regulated following paraquat exposure (logFC 4..00; P: 1.4e−4; adjusted P: 1.9e−2) and is believed to be involved in cell growth^33^ and vesicular trafficking^34^, and is associated with brain neoplasm in the literature. Taken together, the data portray wide-ranging pathway-dependent and compartment-dependent consequences of paraquat exposure on the cardiac proteome, and nominate a number of novel acute response proteins for further characterization.

### Changes in subcellular fraction localization in acute oxidative stress

**Figure 4:**
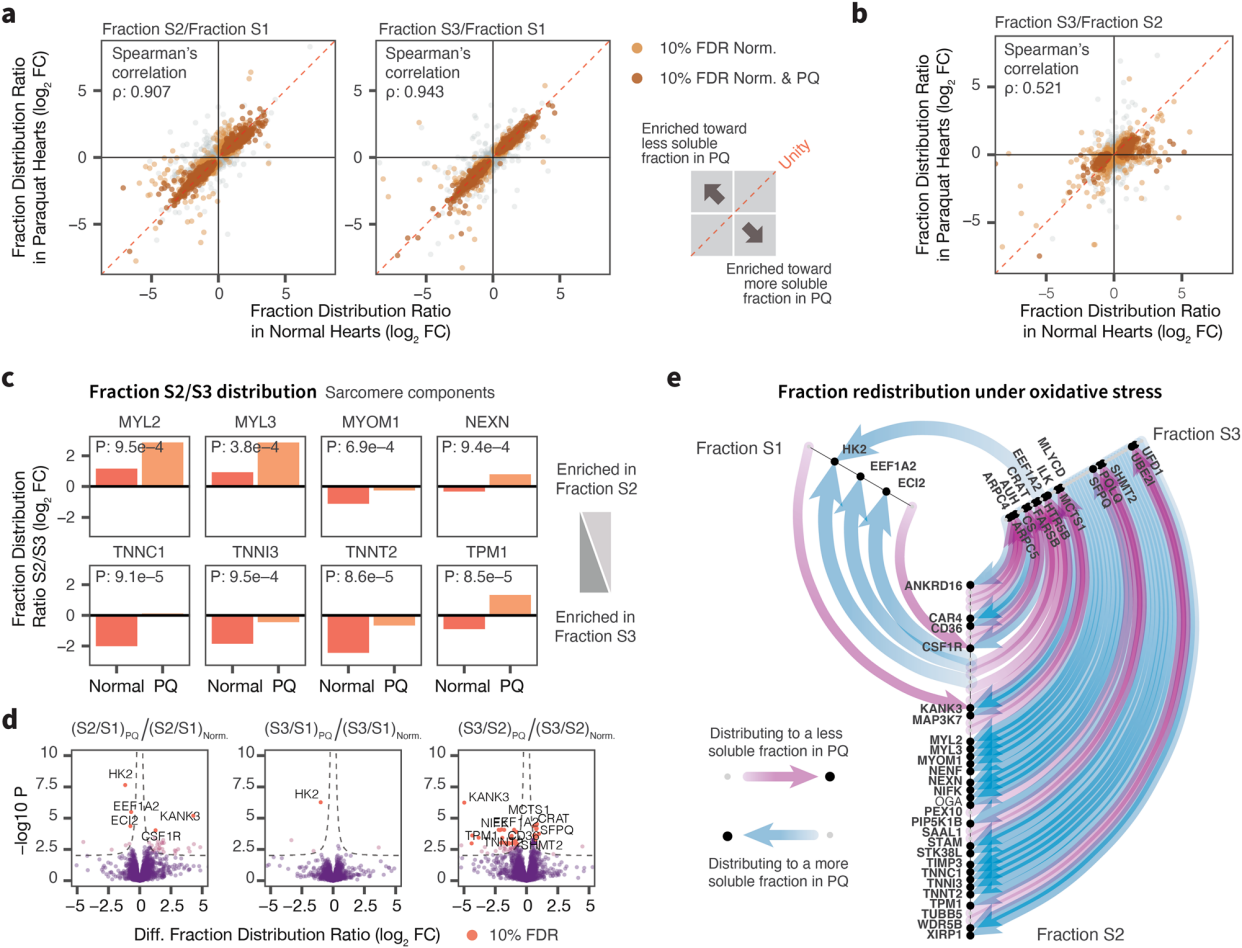
Protein fraction redistribution upon oxidative stress. (**a**) The majority of proteins showed consistent fraction localization in normal (x) vs. stressed hearts (y) when comparing the S2 or S3 fraction to the more soluble S1 fraction (Spearman’s correlation $\rho$: 0.907 and 0.943, respectively), but a subset of proteins showed evidence of preferential distribution to more soluble or less soluble fractions upon stress. Each data point is a protein; color: significant differences in protein abundance across fractions in normal (light brown) or both normal and stressed hearts (dark brown). (**b**) Correlations in relative protein distribution between the S2 and S3 fractions in normal vs. stressed heart (Spearman’s correlation $\rho$: 0.521). (**c**) Bar charts of fraction S2/S3 abundance ratios showing a number of sarcomeric proteins with preferential redistribution from the S3 to the S2 fraction in paraquat (PQ) stressed hearts over normal hearts. Numbers: t-test P values. (**d**) Volcano plots showing significantly redistributed proteins in stressed hearts (x: log2 FC; y: − log10 P). Red data points are significantly different at 10% FDR in limma; up to 10 of the top significant data points are labeled. (**e**) Flow diagram showing redistribution of proteins in stressed hearts among the three subcellular fractions at 10% FDR. Nodes: proteins distributed among the axes representing 3 subcellular fractions; purple edges: redistribution to a less soluble fraction; blue edges: redistribution to a more soluble fraction.

Myofibrillar disarray and alterations in cytoskeleton dynamics are commonly observed in oxidative damage models. To assess whether we could detect this process or other redistribution of proteins from the proteomics data, we examined whether some proteins can be observed to have differential subcellular fraction distribution in normal vs. paraquat treated hearts. We saw that overall the subcellular fraction distribution between the S2 and S1 fractions, and between the least soluble S3 and S1 fractions, are highly correlated in normal and paraquat samples (Spearman correlation coefficient $\rho$: 0.907 and 0.943), indicating the overall fractionation process is reasonably reproducible (Fig. 4a). However, there was also a small subset of proteins that appeared to show altered subcellular distribution between the partially overlapping S2 and S3 fractions ($\rho$: 0.521) (Fig. 4b). We therefore performed a direct comparison of changes in protein ratios between the S2 and S3 fractions in paraquat-stressed hearts over the S2-to-S3 ratio in the normal heart of using the linear model in limma. Among the proteins with significant differences are a number of sarcomeric proteins including myosin and troponin (Fig. 4c).

A question is whether we could distinguish translocation from differential expression. A plausible alternative explanation for the increased ratios of S2/S3 for sarcomeric proteins in paraquat treated hearts would be a rapid increase in the expression of sarcomeric genes, coupled with changes in fractionation efficiency; however, given these proteins are abundant and have long half-life in the mouse heart^35^, we believe massive changes (e.g., doubling in proteome size) within 24 h is unlikely and the more parsimonious explanation of the data would be that it is consistent with the dissolution of myofibrils coupled with appearance in the more soluble fraction. Furthermore, we likewise observed a small but significant number of potential translocation events including prominently hexokinase 2 (HK2) (Fig. 4d). HK2 is known to translocate between the cytosol and mitochondria in the heart following various pathological stimuli^36,37^; thus the result is consistent with at least some of the fraction relocalization proteins representing bona fide spatial translocation. Overall, we observed a complex redistribution of 45 proteins across the subcellular fractions during acute oxidative stress (Fig. 4e). Immunoblot experiment corroborates an apparent redistribution of HK2 from the S2/S3 fraction to the soluble S1 fraction upon oxidative stress, which is not observed to the same extent in the hexokinase 1 (HK1) isoform (Supplementary Fig. S5a,b). Although further establishing the cellular localizations of the proteins will require a different experimental design such as using differential ultracentrifugation, our results nevertheless highlight the opportunity to observe potential protein translocation events as a dynamic aspect of the acute phase proteome response to oxidative stress.

### Responses to global and mitochondria-targeted antioxidant treatments

**Figure 5:**
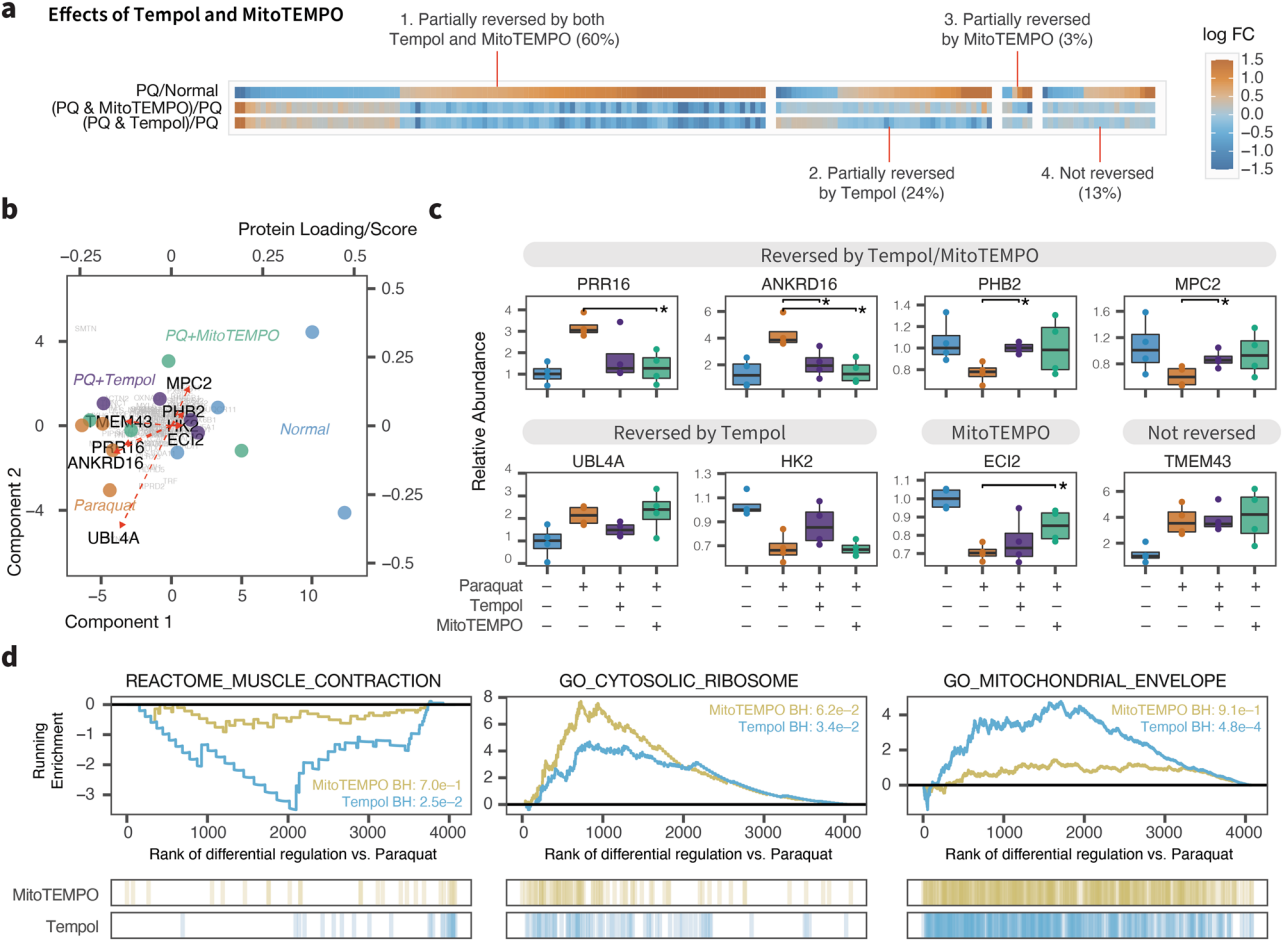
Partial reversal of proteome remodeling by antioxidants. (**a**) Heat map showing the fold changes of 173 cardiac proteins with apparent changes in abundance following paraquat (PQ) treatment ($\hbox {P} < 0.01$; $\vert$logFC$\vert$ $\ge 0.3$) and their fold changes when comparing co-treatment with either antioxidant Tempol or MitoTEMPO with paraquat to paraquat only. The majority of proteins showed partial reversal to the baseline. (**b**) First two components and overlaid protein loadings in linear discriminant analysis between normal, paraquat, and paraquat co-treated with either antioxidants. (**c**) Box plots showing relative abundance of selected proteins, in normal, paraquat, paraquat + Tempol, and paraquat + MitoTEMPO hearts. $^*$t-test $\hbox {P} < 0.05$. (**d**) Comparison of member gene distribution of selected gene sets among proteins ranked by differential abundance in MitoTEMPO + Paraquat vs. Paraquat (yellow) or Tempol + Paraquat vs. Paraquat. Tempol appeared more effective in reverting Paraquat-mediated mitochondrial and muscle proteins whereas MitoTEMPO had a more pronounced effect on ribosomal proteins. x axis: protein rank from highest to lowest log fold change. y axis: running enrichment score. Vertical line segments denote ranks of proteins belonging to the gene sets. Numbers: adjusted P value, parametric gene set enrichment analysis.

To assess whether antioxidant treatment could rescue the detected proteome perturbations, we compared protein expression between paraquat stressed hearts and antioxidant treatments. Mice in the antioxidant treatment groups were administered with either the cytosolic antioxidant Tempol (4-hydroxy-TEMPO or 4-hydroxy-2,2,6,6-tetramethylpiperidin-1-oxyl) (2 mM in drinking water, ad libitum; 24 h)^38^ or its triphenylphosphonium-conjugated lipophilic variant MitoTEMPO which localizes to the mitochondrion^39^ (0.7 mg/kg, i.p.). Considering an arbitrary set of proteins with apparent upward or downward trends following paraquat exposure ($\hbox {P} \le 0.01$; $\vert$logFC$\vert$ $\ge 0.3$), we found that the majority of these proteins reverted toward baseline, including 84% in the Tempol group and 63% in MitoTEMPO group. However, the reversal was mostly partial in magnitude and few reached statistical significance on an individual protein level after multiple-testing correction. Overall, approximately 60% of differentially expressed proteins showed some sign of reversal toward the baseline in both antioxidant groups whereas about 13% of proteins showed no evidence of reversal in our threshold (Fig. 5a).

We also observed evidence that some protein changes were preferentially rescued by one antioxidant over the other. To investigate further, we used a linear discriminant analysis to identify proteins with high weights in discriminating between normal, stressed, and antioxidant treated samples (Fig. 5b). We found that whereas a number of protein changes including PRR16, ANKRD16, PHB2, MPC2 were rescued by both Tempol and MitoTEMPO, UBL4A and HK2 were more reversed in Tempol, and other proteins including TMEM43 were not reversed (Fig. 5c). Subtle differences between Tempol and MitoTEMPO were likewise observed at the pathway level (Fig. 5d). While further investigations will require comparisons of doses, delivery methods, and pharmacokinetics, the data here hint at potential qualitative differences between Tempol and MitoTEMPO, and identify stress-responsive proteins that are not reversed by antioxidant treatments which could be important for the interpretation of antioxidant rescue experiments.

### Contrast with a non-cardiac proteome under paraquat

Finally, we performed a secondary experiment to evaluate how the observed proteome responses in the heart compare with the global response to paraquat in the animal. We collected the lungs from the cadavers of identical animals and subjected them also to isotope labeling tandem mass spectrometry analysis (Supplementary Fig. S6a,b). Both the lung and the heart are high energy-demand tissues that are susceptible to oxidative damage, and previous work has identified the lung to be among the tissues with highest accumulation of paraquat. We therefore examined whether the proteome responses in the lung to paraquat treatment mimicked those in the heart.

At 10% FDR, we found a robust up-regulation of PDK4 (logFC: 3.4; P: 3.1e−4), CAT (logFC: 1.2; P: 2.3e−4), and HMOX1 (logFC: 5.7; P: 2.2e−4) in the lung, indicating paraquat also induced an acute oxidative stress response in the lung of the examined animals. Comparison of organ-specific responses is made challenging by the different proteome composition and dynamic range of concentration between the organs, and moreover, each tissue may be differentially sensitive to paraquat or react in different time scales. Nevertheless, we were surprised to find that the fold-changes of proteins commonly quantified in both the heart and the lungs following paraquat treatment showed little to no correlation to each other ($\rho$: 0.097) (Supplementary Fig. S6c), even when considering only proteins that were nominally differentially regulated in the heart (limma $\hbox {P} \le 0.01$) ($\rho$: 0.22). We observed a number of differentially regulated proteins in the lung that were not changed in the heart, including mitochondrial fission factor (MFF) (lung logFC: 2.03, P: 2.7e−4, adjusted P: 8.7e−2; heart logFC: 0.00, P: 0.99) and glypican 1 (GPC1) (lung logFC 4.5, P: 4.7e−8, adjusted P: 1.6e−4; heart logFC: − 0.01, P: 0.99), as well as annotations including transition metal ion homeostasis (Supplementary Fig. S6d). The lung results and data set may support future investigations into cardiac-specific oxidative stress response elements.

## Discussion

We report here a deep proteomics profiling data set and results on the global protein expression changes of an acute oxidative stressor on the heart using large-scale isotope labelling tandem mass spectrometry. The results presented here and the accompanying data set may avail ongoing efforts to understand how the heart responds to oxidative stress and identify intervention targets. In particular, we identified global changes in metabolism, antioxidant proteins, and proteostasis pathways that are consistent with a complex cardiac response to oxidative stress at the proteome level.

Previous in vitro work in human cervical cancer cells has revealed comprehensive proteome changes under oxidative stress including rerouting of energy metabolism and activation of integrated stress response pathways^8^. Our results here likewise show that there is a prominent metabolic component accompanying oxidative stress response in vivo, and that this metabolic change in paraquat-injured hearts is likely effectuated in part through the activation of PDK4 in the mitochondria. Moreover, our data reveal a number of proteome features not previously highlighted in in vitro experiments, including an indication of sarcomere disarray reflected by an apparent increase in muscle contraction proteins, as well as a redistribution of proteins between cardiac subcellular fractions. Secondly, the in vivo investigation here allowed a contrast of oxidative stress between tissues. Both the heart and the lungs are highly susceptible to oxidative damage and acute paraquat exposure has been widely used in animal models of oxidative stress in both organs. In the lungs of identical animals, we found a robust up-regulation of HMOX1 and PDK4, as well as a general decrease in mitochondrial and metabolic proteins (Supplementary Fig. S6c,d). Surprisingly however, the overall correlation in protein fold changes between the tissues is poor, which is likely in part contributed by both cardiac changes in sarcomeric proteins as well as lung-only response factors. The data from the paraquat-exposed lungs further corroborate that PDK4 likely plays a central role to metabolic control in oxidative stress response. However, as the PDK4 isoform of pyruvate dehydrogenase kinase is not ubiquitously expressed across tissues, it would be of interest to assess whether similar metabolic responses exist in other tissues in paraquat-treated animals.

With the experimental design incorporating subcellular fractionation, we found that paraquat preferentially induced changes in one fraction (S2) over the others in the heart. Differential detergent lysis is a common method to resolve partially overlapping protein fractions that correspond in part with specific cellular compartments^40^. It is now appreciated that different cellular compartments have distinct requirements and mechanisms for maintaining protein integrity^8,41^. The results here corroborate that paraquat primarily affects proteins in mitochondria and endoplasmic reticulum enriched compartments in the heart. This compartment difference was also investigated through treating the animals before paraquat exposure with Tempol or simultaneously with MitoTEMPO (Fig. 5). Both Tempol and MitoTEMPO are redox cycling nitroxides, with MitoTEMPO additionally possessing a lipophilic triphenylphosphonium moiety that promotes its accumulation in the mitochondria. Notwithstanding the mitochondrial respiratory chain being thought to be the site of reactive oxygen species generation after paraquat exposure, we found that both Tempol and MitoTEMPO treatments led to clear trends of partially reverting the paraquat-mediated changes of both cytosolic and mitochondrial proteins. There are also subtle differences in the response to both antioxidants with Tempol treatment paradoxically showing a greater reversion of mitochondrial protein changes, but the cause of this difference is not investigated here.

Surprisingly, we also found evidence that some proteins may redistribute in fractions upon paraquat challenge. These results therefore indicate that spatial dynamics may be a component of proteomic features during the acute stress response and echo the increasing number of reports that demonstrate proteome-wide spatial redistribution upon a variety of stimuli^42^. A caveat of the current experimental design is that differential lysis provides a relatively crude method to separate cellular compartments based partly on membrane composition and compartment solubility, which could differ across cells and tissues. In fact, analysis of the proteomics results from different fractions in the heart shows that the cellular components in detergent fractions can overlap partially, and suggests direct mass spectrometry evidence should be sought when similar methods are applied to fractionate different cell type or samples. Future work may apply orthogonal methods such as ultracentrifugation in conjunction with detergent based methods to follow up on spatial proteomics findings and identify additional translocalization candidates.

Finally, the mechanisms behind the observed differential expression changes are not investigated in this study and could be an important future direction. The abundance of a protein pool may be controlled by both the protein’s synthesis rate and its degradation rate. In some instances where a protein requires fast abundance control in response to abrupt cellular and environmental changes, proteolysis provides a mechanism to quickly adjust protein abundance without requiring transcriptional regulation. This is evidenced by the activation mechanism of NRF2 in response to oxidative stress as well as other examples including PINK1 to mitochondrial membrane potential and HIF1a to oxygen level. Protein damage and unfolding may prompt targeted degradation, whereas autophagy is another prominent protein removal mechanism that is well described to respond to oxidative stress^43,44^. Future work might examine if the observed changes in metabolic and mitochondrial proteins were effectuated in the heart by autophagy of specific cellular components.

## Methods

### Animal models and tissue collection

Animal experiments were approved and performed in accordance with the Institutional Animal Care and Use Committee (IACUC) guidelines at University of Colorado Denver/Anschutz Medical Campus. Twelve-week old male wild-type C57BL/6J mice were purchased from Jackson Laboratories (Bar Harbor, ME, USA) and housed in accordance with guidelines set by the National Institutes of Health (NIH) for the Care and Use of Laboratory Animals. The protocol for animal use was approved by the Animal Care and Use Committee of the University of Colorado School of Medicine. Animals were held in a temperature-controlled environment on a 12-h light/dark cycle and supplied with pellet chow and water ad libitum. In the first animal experiment, mice were randomized into six treatment groups (n = 4 per group each) and received either Dulbecco’s phosphate buffered saline (DPBS) ($200 \, \upmu \hbox {L}$, i.p.) or paraquat (*N*,*N*$^\prime$-dimethyl-4,4$^\prime$-bipyridinium dichloride) at three doses (10, 50, 75 mg/kg i.p.) and examined after 24 h. One group was examined after 48 h of 50 mg/kg paraquat i.p, and the last group received 2 mM Tempol (4-hydroxy-2,2,6,6-tetramethylpiperidin-1-oxyl) in drinking water for 24 h ad libitum prior to paraquat (50 mg/kg i.p.) treatment and examined after 24 h post-paraquat. Chemicals were purchased from Sigma unless specified.

In the second experiment, mice were randomized into four treatment groups (n = 5 per group). Mice in vehicle and paraquat exposure groups received either DPBS ($200 \, \upmu \hbox {L}$, i.p.) or paraquat (50 mg/kg, i.p.) for 24 h, respectively. Mice in the antioxidant treatment groups were administered with Tempol (2 mM in drinking water, ad libitum) or its triphenylphosphonium-conjugated lipophilic variant MitoTEMPO (0.7 mg/kg, i.p.) for 24 h prior to administration with paraquat (50 mg/kg, i.p.) for 24 h. Paraquat and MitoTEMPO solutions were prepared in DPBS. One group of paraquat-treated animals was euthanized prior to the experimental end-point; and the remaining four replicate groups were used in the experiments. Following exposures, mice were euthanized by CO_2_ displacement followed by cervical dislocation, and the heart and lungs were harvested. The harvested tissues were rinsed in PBS, snap-frozen immediately in liquid nitrogen, and stored at − $80^{\circ }\hbox {C}$ until analysis.

### Compartmental protein extraction and digestion

To extract proteins from subcellular fractions of the mouse tissues, harvested tissues were washed with PBS, blotted dry, and weighed, then homogenized using an Omni tissue homogenizer (Omni International, Kennesaw, GA, USA) using 3 pulses of 20 s each at setting 4. Subcellular fractions were extracted using differential lysis and solubilization with a commercial Millipore Compartmental Protein Extraction Kit (Millipore) according to manufacturer’s instructions. Briefly, compartments S1, S2, and S3 were attained through detergents supplied as buffer C (KCl, glycerol, sodium orthovanadate), buffer N (NaCl, glycerol, sodium orthovanadate), and buffer M (KCl, sucrose, glycerol, sodium deoxycholate, NP-40, sodium orthovanadate). Following extraction, total protein concentration from each fraction was determined by bicinchoninic acid assay (Thermo Pierce). $150 \, \upmu \hbox {g}$ of protein was digested on a molecular weight filter. Briefly, samples were placed on 10 kDa MWCO polyethersulfone filters (Thermo Pierce), denatured with 8 M urea, and buffer exchanged into 100 mM triethylammonium bicarbonate (TEAB). Samples were reduced ($55\;^{\circ }\hbox {C}$, 30 min) with 3 mM Tris(2-carboxyethyl)phosphine hydrochloride (TCEP-HCl) (Thermo Pierce) while shaking at 600 rpm in a thermomixer (Eppendorf). Samples were alkylated with 9 mM iodoacetamide ($22\;^{\circ }\hbox {C}$, 30 min) in the dark with 600 rpm shaking, then digested on-filter with sequencing-grade trypsin (Promega) at a ratio of 50:1 (w/w) for 16 h at $37\;^{\circ }\hbox {C}$, shaking at 600 rpm. Post-digestion peptide concentration was determined using a Pierce quantitative colorimetric peptide assay kit (Thermo Pierce) then labeled using tandem mass tag isobaric stable isotope labels (Thermo TMT10plex; lot TA260585).

### Liquid chromatography and tandem mass spectrometry

We used a block multiplexing approach to design the randomization scheme and assign the comparison group samples to tandem mass tag channels. For each tissue, samples from all treatment groups and subcellular fractions were randomized to one of ten channels in one of six blocks using the RAND function in Excel with no control over randomization seed (Supplementary Fig. S1). Biological replicates within the same subcellular compartments were assigned to the same blocks where possible for batch correction. For accurate comparisons across blocks, two identical internal reference standards were also generated by combining $20 \, \upmu \hbox {g}$ of peptides pooled from nine representative samples within a labeling block, and randomized to two channels in each block. TMT10plex labeling for each block was performed according to manufacturer’s instructions. Briefly, TMT10plex reagents were reconstituted in $41 \, \upmu \hbox {L}$ of acetonitrile, combined with $10 \, \upmu \hbox {g}$ of peptide, and incubated for 1 h at $22\;^{\circ }\hbox {C}$. The labeling reaction was quenched by incubation with $8 \, \upmu \hbox {L}$ of 5% hydroxylamine in 100 mM TEAB for 30 min at $22\;^{\circ }\hbox {C}$, shaking at 600 rpm. The labeled peptides were subsequently combined, dried by vacuum centrifugation, and reconstituted in $300 \, \upmu \hbox {L}$ of 0.1% trifluoroacetic acid in LC-MS grade water. Samples were then subjected to offline reversed phase fractionation (8 fractions) using a Pierce High-pH Reversed Phase Fractionation Kit (Thermo Fisher) according to the manufacturer’s instructions for fractionation of TMT-labeled samples.

Liquid chromatography and tandem mass spectrometry were used to analyze the fractionated labeled peptides as previously documented^45^. Second dimension petpide separation was performed with online reversed-phase liquid chromatography using an Easy-nLC 1200 nanoflow ultrahigh-pressure liquid chromatography (UPLC) system (Thermo Scientific), which was connected to a Q-Exactive HF hybrid quadrupole orbitrap mass spectrometer (Thermo Scientific) through an EasySpray interface (Thermo Scientific). Chromatographic separation was conducted on an EasySpray PepMap C18 column ($3 \, \upmu \hbox {M}$ particle size; 100 Åpore size; $75 \, \upmu \hbox {m}$ internal diameter $\times 150 \, \hbox {mm}$ length; Thermo Scientific). We injected up to $3 \, \upmu \hbox {L}$ peptide samples on column using an autosampler. The nano-UPLC system operated at 300 nL/min, with a solvent gradient as follows: 0–105 min, 0–40% B; 105–110 min, 40–70% B; 110–115 min, 70–100% B; hold for 5 min. Solvent A is 0.1 % formic acid in HPLC-grade water (v/v); solvent B is 80% acetonitrile and 0.1% formic acid in HPLC-grade water (v/v). Characteristic mass spectrometry settings are as follows: MS1 scans at 60,000 resolving power, in profile mode, positive polarity, mass range 300–1650 *m/z*; 30 s dynamic exclusion; maximum injection time 20 ms; lock mass; automatic gain control target of $3 \times 10^6$. Fragment ion (MS2) scans were acquired for the top 15 parent ions using monoisotopic peak selection at 60,000 resolving power; automatic gain control target of $2 \times 10^5$; isolation window 1.4 *m/z*; maximum injection time 100 ms; typical normalized collision-induced dissociation energy (NCE) 32.

The Thermo raw spectrum files were converted to the open source .mzML format^46^ using ThermoRawFileParser v.1.2.0^47^ with the options (-g -f 2). MS2 spectra were searched using SEQUEST algorithm implemented in Comet (v.2019.01 rev.4)^48^ against a .fasta database Uniprot/SwissProt *mus musculus *database accessed on 2020-03-19 (17,033 target entries)^49^. Comet search parameters followed conventional standards including: peptide_mass_tolerance=10, peptide_mass_units=2, isotope_error=3, num_enzyme_termini= 1, allowed_missed_cleavage=2, fragment_bin_tol = 0.02. Variable methionine oxidation (M + 15.9949 Da), static cysteine carbamidomethylation (C + 57.021464 Da), and static tandem mass tag addition to lysine or the peptide N-terminus (nK + 229.16293 Da) were specified. Peptides were reranked and assigned with confidence of identification using the Crux/Percolator v.3.2^50^ followed by protein inference, with peptides accepted for identification under 0.01 Percolator q-value.

### Proteomics quantification and additional statistical analysis

As the Crux/Percolator pipeline does not come with a native isobaric label quantification tool, we wrote a Python 3 tool, py-tmt-quant, to integrate the label channel intensities of each MS2 spectrum in the experiment. The software tool reads in the supplied Percolator result file and a directory of corresponding mzML files using the pymzml library^51^, and returns an appended search result file with the channel intensities. It is compatible with MS2-level tandem mass tag experiments using TMT 0, 2, 6, 10, 11, and 16-plex tags and allows user-defined mass tolerance for integration (see Code Availability). Using py-tmt-quant we integrated the centroid peak intensity within 10 ppm of each channel m/z. For each peptide-charge combination, only the PSM with the best Percolator score per fraction is integrated. We corrected for isotope impurities using the contamination matrix from the manufacturer (Supplementary Fig. S1b). PSMs were filtered to exclude peptides from common contaminants (albumin, hemoglobins, and keratins), those without any TMT channel intensities, and those with no internal reference standard channel intensity, as well as those with low TMT channel intensities below 10% of total spectral intensity.

We performed sample-level dye correction through column sum normalization. We then performed blockwise correction using the ComBat batch correction function implemented in the sva package (v.3.35.2)^52^ in R (v.3.6.3)/Bioconductor (v.3.10)^53^. Spectrum-level variances were normalized using the median values of the internal reference standard channel intensities across each block, and finally the effective total intensities were normalized using trimmed means of m-values with singleton pairing as implemented in the edgeR package (v.3.29.1)^54^ in R/Bioconductor (Supplementary Fig. S1c). Statistics on differential protein expression were performed using the moderated t-test implemented in the limma package (v.3.43.5)^55^ correcting for duplicate correlations of subcellular fractions from identical animals, followed by the Benjamini-Hochberg procedure^56^ to correct for multiple testing and calculate false discovery rates. Additional analysis on protein function was performed with the aid of StringDB^57^, Pubpular^58^, PGSEA^59^, Reactome^60^, and Enrichr^32^. Additional visualizations were created in R v.3.6.3. with the aid of the ggplot2, hiveR^61^, and gganatogram^62^ packages.

### Immunoblotting

Additional validation experiments were performed using immunoblots. Protein lysates (50 g per lane), as determined by bicinchoninic acid assay (BCA) (Pierce) from two biological replicate animals each from compartments S1, S2, and S3 for vehicle and 50 mg/kg paraquat hearts were denatured by boiling and resolved on 12% sodium dodecyl sulfate-polyacrylamide gels. Proteins were then transferred to polyvinylidene fluoride membranes and stained with Ponceau S (Sigma). After de-staining, membranes were blocked in 5% bovine serum albumin at $22\;^{\circ }\hbox {C}$ for 2 h and then probed with either anti-Hexokinase II (Cell Signaling Technology, cat. #2867, 1:1000 dilution) or the negative control anti-Hexokinase I (Cell Signaling #2024, 1:1000 dilution) at $22\;^{\circ }\hbox {C}$ for 2 h, followed by anti-rabbit IgG, HRP-linked secondary antibody (Cell Signaling #7074, 1:1000 dilution) at $22\;^{\circ }\hbox {C}$ for 2 h. Pierce ECL Plus western blotting substrate (Thermo) was used to detect targeted proteins using Typhoon 9400 Imager (Molecular Dynamics). The ImageJ software (National Institutes of Health, Bethesda, MD, USA)^63^ was used to quantify the intensity of the bands of interest and normalized to Ponceau Stain^64^. Statistical tests on the densitometry values were performed using Welch’s t-test in R v.3.6.3.

## Supplementary information

Supplementary Information.

## Data Availability

Raw mass spectrometry data have been deposited to the ProteomeXchange Consortium via the PRIDE^65^ partner repository with the dataset identifier PXD020199. The latest version of py-tmt-quant (v.0.3.0) is accessible on https://github.com/Lau-Lab/pytmt.
