# Supplementary material for: Proteomic signatures of acute oxidative stress response to paraquat in the mouse heart
Source: Sci Rep. 2020 Oct 28;10:18440. doi: 10.1038/s41598-020-75505-8 (PMC7595225; doi:10.1038/s41598-020-75505-8)
Supplement: Supplementary file 1 — Supplementary Information. [file 41598_2020_75505_MOESM1_ESM.pdf]

Supplementary Materials

Proteomic signatures of acute oxidative stress response to paraquat in the mouse heart

Vishantie Dostal[1,2,3,&] , Silas D. Wood[1,2,3,&] , Cody T. Thomas[1], Yu Han[1,2,3], Edward Lau[1,3], Maggie P.Y. Lam[1,2,3]*

[1]Departments of Medicine/Cardiology and [2]Biochemistry & Molecular Genetics
[3]Consortium for Fibrosis Research & Translation
University of Colorado Anschutz Medical Campus, Aurora, CO 80045, USA.

* Corresponding Author: Maggie P. Y. Lam
University of Colorado, Anschutz Medical Campus
12700 E. 19th Avenue
Aurora, CO 80045
E-mail: maggie.lam@cuanschutz.edu

[&] These authors contributed equally to this work

Competing interests
The authors declare no competing interests.

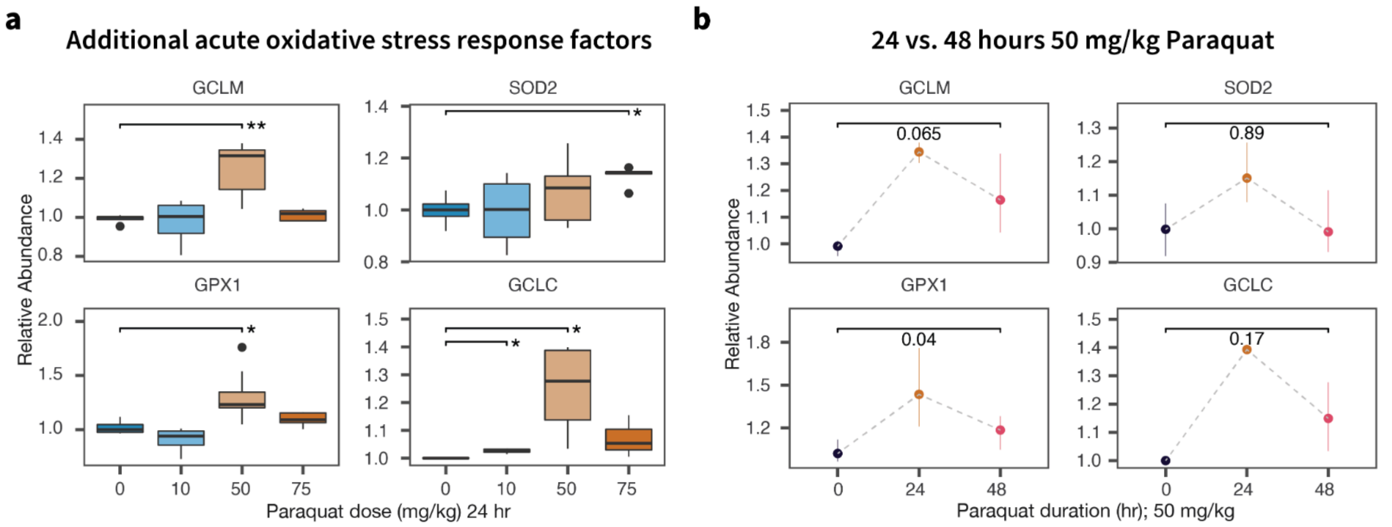

Figure S1 | Paraquat induced changes of additional oxidative stress response proteins. **a.** Protein abundance of GCLM. GCLC, SOD2, and GPX1 at three doses of paraquat for 24 hours; $*$: t-test $P < 0.05$; $**$: 0.005. **b.** Comparison between 24 hr and 48 hr treatment of 50 mg/kg paraquat; Numbers: t-test P values.

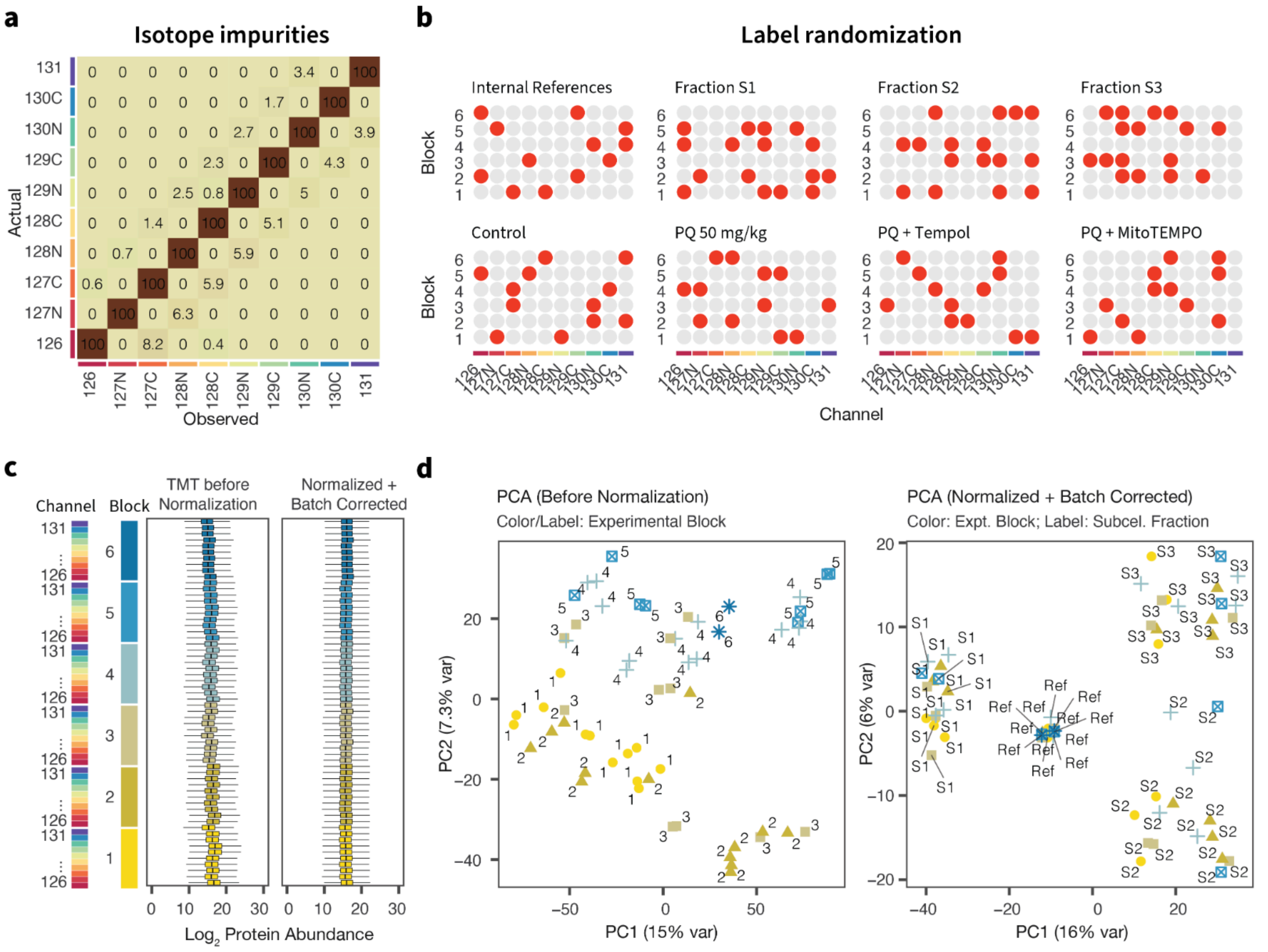

Figure S2 | Isotope labeling tandem mass spectrometry analysis of normal and stressed hearts. **a.** Isotope impurity matrix for the tandem mass tag channels supplied by the manufacturer. **b.** Label assignment of samples in 6 experimental batches. Labels were randomized using a random number generator in Excel. **c.** Distribution of log label abundance before and after normalization and batch correction. **d.** Principal component analysis showing preferential grouping of channel intensities across experimental batches prior to normalization, and correction after.

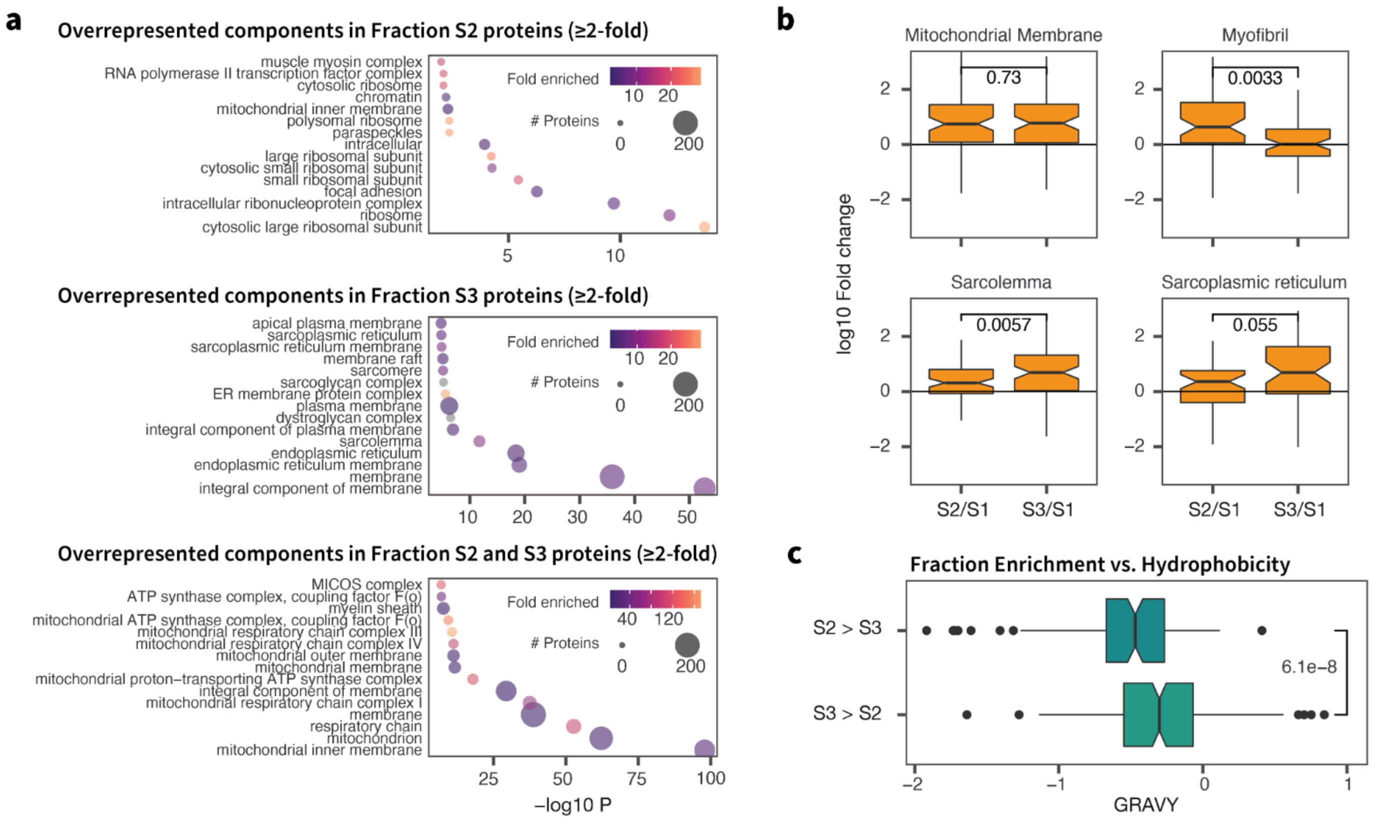

Figure S3 | Properties of proteins enriched in subcellular fractions. **a.** Top 15 significantly enriched Gene Ontology cellular compartment terms in proteins in the S2 (top); S3 (middle); and both S2 and S3 fractions over S1. Data point size denotes number of proteins associated with a term; color denotes fold enrichment of term representation over background; x: –log P of hypergeometric test. **b.** Box plots showing relative enrichments in S2 and S3 fractions over S1 of proteins in the mitochondrial membrane, myofibril, sarcolemma, and sarcoplasmic reticulum. **c.** Grand average of hydrophobicity (GRAVY) scores of proteins that preferentially enrich in S2 vs. S3 fraction, indicating the S3 fraction contains more hydrophobic proteins.

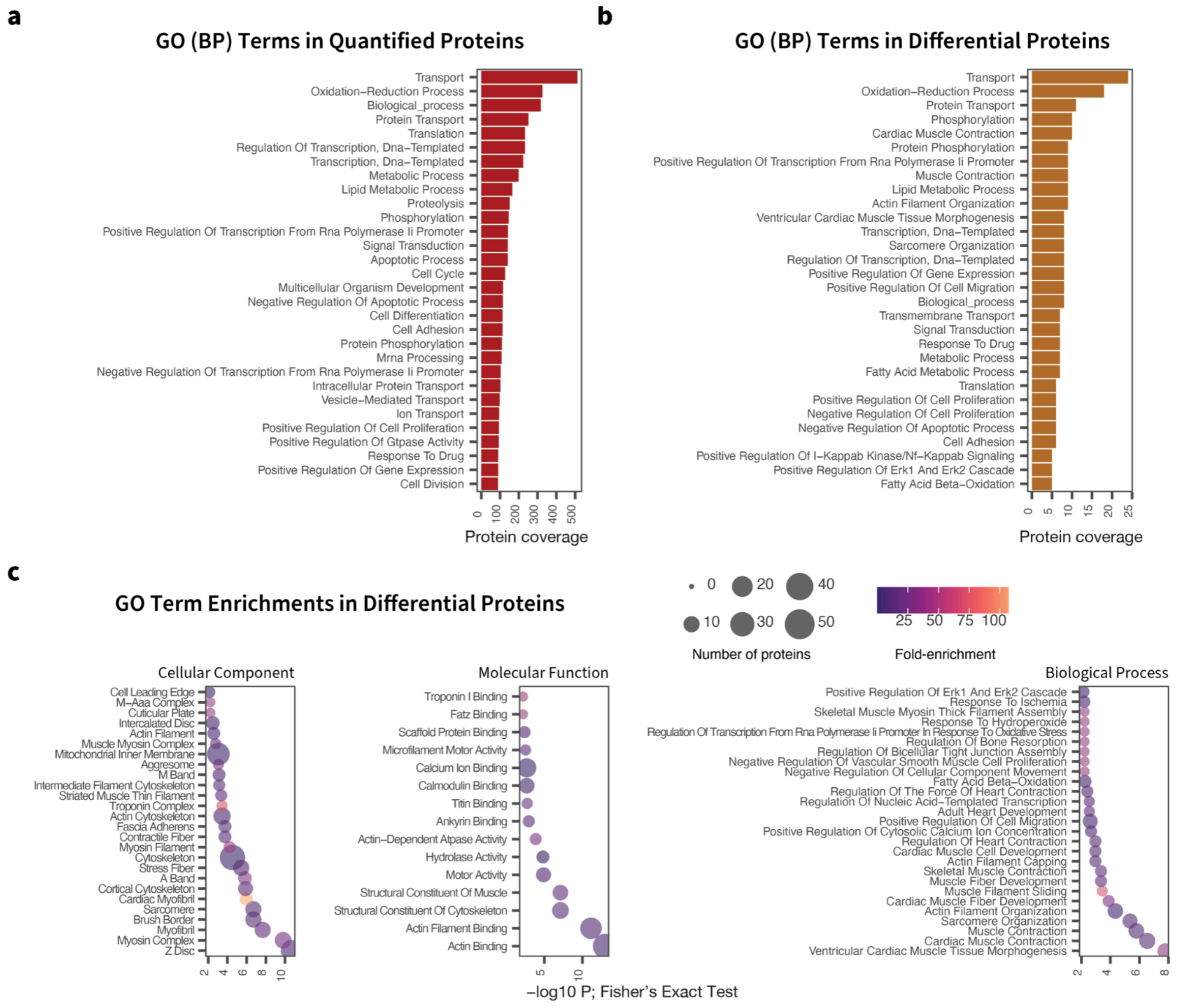

Figure S4 | Annotated gene functions in acute oxidative stress comparisons. **a.** Bar charts showing the protein coverages of top represented Gene Ontology (GO) Biological Processes (BP) terms among quantified proteins in the data set. **b.** Top represented GO BP terms among differentially expressed proteins (Fraction S2, 50 mg/kg paraquat vs. vehicle). **c.** GO annotation term over-representation analysis among differentially expressed proteins (Fraction S2, 50 mg/kg paraquat vs. vehicle). Top enriched terms for Cellular Component, Molecular Function, and Biological Process aspects of GO are shown. x: –log 10 P value, Fisher's Exact Test. Color: Number of proteins in foreground. Color: Fold-enrichment over background (all quantified proteins).

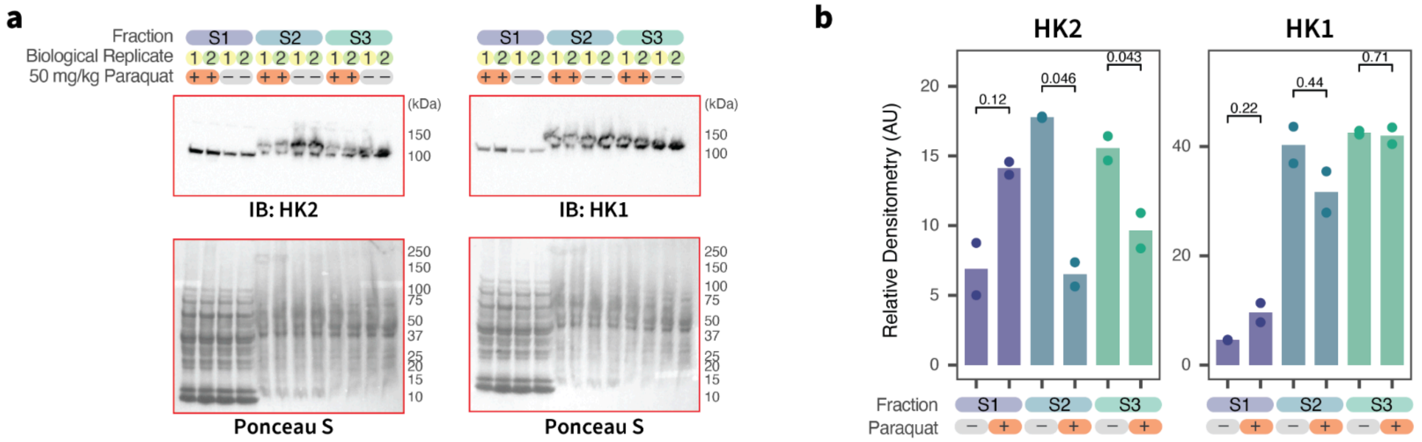

Figure S5 | Uncropped immunoblots of hexokinase subcellular distributions under oxidative stress. **a.** To corroborate the proteomics data, we performed immunoblots of hexokinase 2 (HK2; left) and hexokinase 1 (HK1; right) across three subcellular fractions (S1, S2, and S3) with or without 50 mg/kg Paraquat. **b.** Densitometry analysis of the immunoreactive chemiluminescent bands in panel a shows that HK2 had increased relative abundance in the soluble fraction S1 with concomitant decreases in the organellar/membrane S2/S3 fractions following paraquat exposure. This effect was subdued in the HK1 isoform with no noticeable decrease in S3 abundance following paraquat exposure. Numbers: P values, t-test. (Uncropped image is included in the supplementary materials file)

Original gel images and ladders included below:

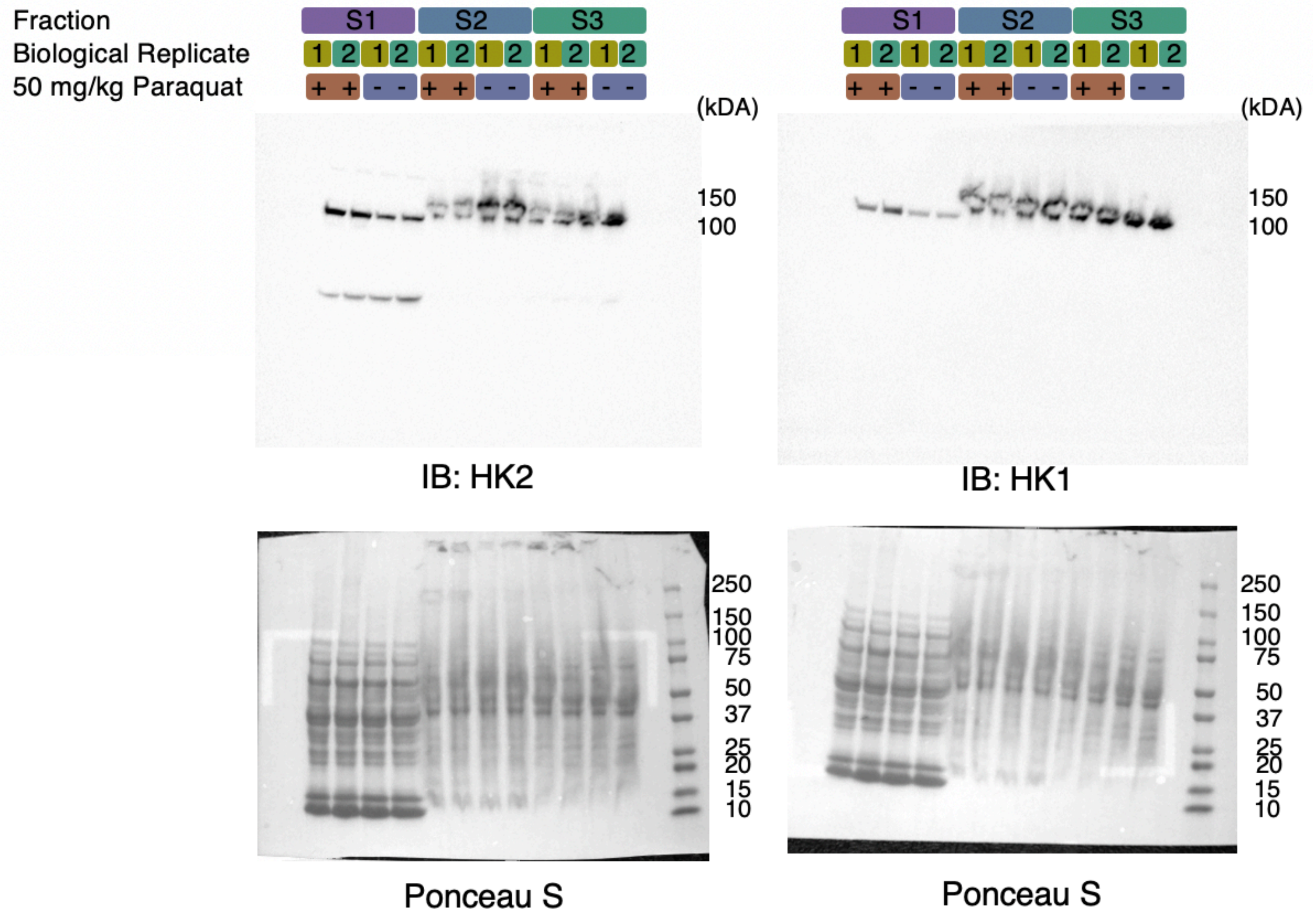

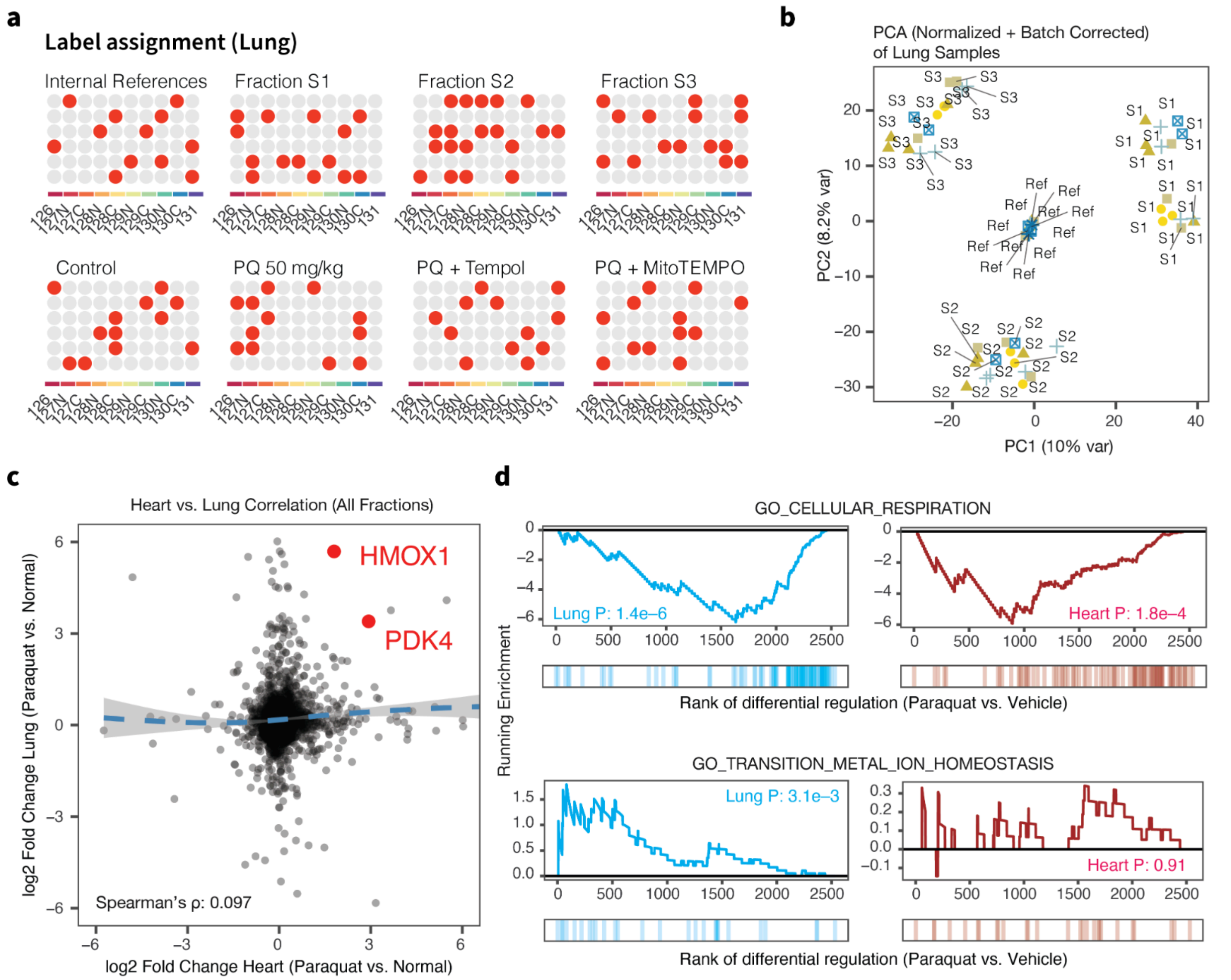

Figure S6 | Isotope labeling tandem mass spectrometry analysis of normal and stressed lungs. **a.** Label assignment of samples across 6 experimental batches. Labels were randomized using a random number generator in Excel. **b.** Principal component analysis showing expected preferential grouping of samples by subcellular fractionation following normalization and batch correction. **c.** Protein responses to paraquat between the heart (x axis) and the lung (y axis) showed modest correlation (pairwise-complete Spearman's rho 0.097). HMOX1 and PDK4 appeared to be robustly induced in both organs (highlighted in red). **d.** Parametric gene set enrichment analysis on ranked log fold changes of proteins in the two organs show similarities and differences. (Top) Respiratory chain proteins were similarly suppressed in both organs, whereas other gene sets including metal ion homeostasis appeared more induced in the lungs than the heart.

Table S1 | List of all quantified proteins in Fractions S1, S2, and S3 from TMT mass spectrometry experiments between 50 mg/kg paraquat and vehicle treated animals. See Excel file via figshare link: dx.doi.org/10.6084/m9.figshare.13020611
